# Profiling the Injuries Sustained by Police Trainees Undergoing Initial Training: A Retrospective Cohort Study

**DOI:** 10.3390/ijerph18147335

**Published:** 2021-07-08

**Authors:** Sally Sawyer, Ben Schram, Rodney Pope, Robin Orr

**Affiliations:** 1 Faculty of Health Sciences and Medicine, Bond University, Gold Coast, QLD 4229, Australia; sally.sawyer@student.bond.edu.au (S.S.); bschram@bond.edu.au (B.S.); 2 Tactical Research Unit, Bond University, Gold Coast, QLD 4229, Australia; rpope@csu.edu.au; 3 School of Allied Health, Exercise and Sport Sciences, Faculty of Science and Health, Charles Sturt University, Wagga Wagga, NSW 2640, Australia

**Keywords:** recruit, cadet, injury

## Abstract

The tasks performed by police officers are unique, varied and can be performed in unexpected situations. Initial police college training is used to prepare new police officers to conduct these tasks and is known to be a time when police trainees are at an elevated risk of injury. The aim of this study was to profile injuries occurring within a national Police Force during initial training to inform injury prevention strategies. Using a retrospective cohort design, point-of-care injury data including injury body site, nature, mechanism, and the activity being performed at the time of injury were provided. A total of 564 injuries were recorded over the 22-month period, with the mean age of recruits reporting an injury being 28.83 years ± 6.9 years. The incidence of injuries ranged across training periods, from 456.25 to 3079 injuries per 1000 person-years with an overall incidence rate of 1550.15 injuries per 1000 person-years. The shoulder was the most injured site (*n* = 113, 20% of injuries), with sprains and strains being the most common nature of injury (*n* = 287, 50.9% of injuries). Muscular stress with physical exercise was the most common mechanism of injury (*n* = 175, 31.0% of injuries) with the activity responsible for the largest proportion of injuries being “unknown” (*n* = 256, 45.4% of injuries), followed by police training (*n* = 215, 38.1%). Injuries appear to be typically joint related—commonly the shoulder—with police training being a primary known activity at the time of injury. Prescreening protocols may be of benefit, and efforts should be made to recruit and train physically resilient trainees. Injuries, whether they occurred pre-enlistment or during training, should be fully rehabilitated prior to the individual’s commencement as a qualified officer.

## 1. Introduction

The tasks performed by police officers are unique, varied, and can be performed in unexpected situations [1]. While these tasks can include predominately sedentary duties, an officer needs to be prepared for high-intensity and physically demanding situations when required [2]. These situations include the apprehension and containment of offenders, crowd control, and assisting in emergency situations [1,2]. These duties are composed of multiple functional requirements, requiring strength, endurance, agility, balance, and power, and are often completed with minimal notice, highlighting the physical nature of law enforcement [3,4]. In order to perform these tasks effectively, officers need to have a sufficient level of physical preparedness to ensure job competency and public safety [5].

The physicality associated with law enforcement tasks and duties increases the risk of injury in this population [6]. Lyons et al. [7] found the incidence of injuries within law enforcement personnel to range from 240 to 2500 injuries per 1000 personnel. Apart from these injuries impacting on the officers personally, injuries also impact the law enforcement agency in which they work, creating financial and workforce challenges [8]. As an example, one Australian Police Force estimates an average cost of recruiting and training to be AUD 85,000 per trainee [9].

Police college training includes aspects of both physical fitness and specific law enforcement skills [3]. These skills consist of defensive tactics, marksmanship training, and the load carriage of body armour and daily accoutrements [10]. Physical training of police trainees optimizes body composition and improves physical performance measures, such as power and aerobic capacity [11,12]. These improvements can improve health status, protect against injury and illness, and improve occupational performance [13]. Conversely, the increase in physical demands required when undergoing this training can place police trainees at a higher risk of injury when compared to their trained colleagues [14,15,16].

Research has shown that fitter trainees are less prone to injury, whereby trainees who have higher physical fitness are more likely to withstand the physical demands imposed by commencing training and the physical stress associated with it [11,17,18,19]. An example is provided by a retrospective study conducted by Knapik et al. [18], investigating injuries and physical fitness during new agent training for Federal Bureau of Investigation trainees. The study found that trainees who had higher fitness levels (measured as push-ups, sit-ups, pull-ups, 300 m sprint, and 1.5 mile run) were less likely to incur injuries during their initial training [18]. Similar findings (using shuttle run and 75 m pursuit assessments) have been confirmed in other police trainee populations [20].

On this basis, injury prevention strategies, which can include fitness training and assessments [19], are typically put in place during initial police training to help mitigate injury risk. Intensity, frequency, and duration of training are all parameters which can be modified to minimize injury risk [21]. For example, Pope et al. [22] reported on changes made to army physical training, including reducing the speed of endurance marches with load, which resulted in a significant decrease in the incidence of pelvic stress fractures in female trainees. While interventions have the potential to mitigate injuries, an initial requirement prior to implementing a risk mitigation strategy is to establish the context of the injuries sustained [23]. The approach to establishing the context of risk is well-used in risk management platforms [24] and has been used in tactical populations for this purpose [25]. A detailed profiling of injuries sustained within a given population allows the application of specific interventions to minimize injury risk in high-risk areas. The aim of this study was to profile the injuries occurring within the New Zealand (NZ) Police during initial training to inform injury prevention strategies.

## 2. Materials and Methods

A cohort study design, using data previously collected and stored prospectively as part of standard New Zealand Police College routine operational practices, formed the basis of this research. The injury data, spanning a 22-month period (from April 2017 to January 2019), were recorded for trainees undergoing initial training at the Police College, with training courses running over a 16-week period. An injury was defined as an accident or incident in an unplanned and unexpected event with undesirable or unfortunate consequences that harmed a worker or other person in the workplace [26]. The injury data extracted for this study included the numbers of injuries sustained, injury body locations, natures of injury, injury mechanisms, and activities being performed at the times injuries occurred. The numbers of trainees undertaking training during each of the 3-month periods covered by the data were also provided by the New Zealand Police College, to allow calculation of injury incidence rates. The Bond University Human Research Ethics Committee granted ethics approval for this study (BUHREC, Research Protocol BS02086). The usual requirement to obtain participant consent for use of the data in the study was waived as part of this approval, as the data was retrospective and nonidentifiable. Gatekeeper approval for use of the data was provided by the law enforcement agency from which these data were drawn.

### Data Analysis

Data were provided in an Excel (Microsoft Corporation, Redmond, WA, USA) spreadsheet, examined by the researchers for accuracy, and manually cleaned to remove duplicates and ineligible records (e.g., staff member) and then imported into SPSS (IBM, Armonk, NY, USA). Raw injury counts and injury incidence rates were calculated for each 3-month period of training covered by the injury data, the latter in terms of injuries per 1000 person-years of training. Raw numbers of each injury type were calculated and expressed as a percentage of overall injuries. Descriptive analyses were then performed to further describe the reported injuries, in terms of counts and proportions of injuries falling within specific categories. Where no information was provided regarding the body part injured, the “free descriptive text” field of the respective injury record was reviewed and, where possible, the injured area was identified and recorded. Where this information could not be determined, it was coded as unknown. To investigate differences in injury presentations across time periods, a chi-square test of homogeneity was performed. Post hoc z-tests with Bonferroni adjustment were subsequently performed to determine where differences, if any, lay. The final time period (21 January 2019–3 February 2019) was excluded from the analysis as this period was not a complete 3-month period.

## 3. Results

Data for 702 reported incidents were provided. From these, 23 entries were removed as they related to injuries suffered by College staff, 65 entries were removed as they were reporting near misses without a physical injury, and a further 50 entries were removed because they were duplicates. A total of 564 injuries reported over the 22-month period remained represented in the injury records after the data were cleaned. The number of injuries reported in each 3-month period for which injury data were provided is shown in Table 1. The incidence of injuries ranged from 456.25 to 3079 injuries per 1000 person-years with an incidence rate of 1550.15 injuries per 1000 person-years overall. The rates of injuries were higher overall in 2018–2019 (1861.99 injuries per 100 person-years) when compared to a comparative period (21 April–20 January) from 2017 to 2018 (1133.71 injuries per 100 person-years). The rates of injuries fluctuated substantially across the 3-month periods. Significant differences (*p* < 0.001) were found between time periods with significantly lower rates over the time period 21 October 2017–20 January 2018, and highest rates generally in the 3-month periods spanning 21 July 2017–20 October 2017 and 21 January 2018–20 January 2019, with the exception of 21 July 2018–20 October 2018 (Table 1).

The mean age of trainees reporting an injury was 28.83 ± 6.90 years, ranging from 18 to 50 years of age. The proportions of injured trainees that were in each age bracket are shown in Table 2. The majority of injured trainees were between 20 and 29 years of age, representing 60% (*n* = 337) of injuries. This was followed by those aged 30–39 years, representing 29% of injuries (*n* = 165). As no data were available regarding proportions of trainees from the total 1660 trainees who were in each age bracket, the incidence of injuries within each age bracket could not be derived.

The body sites of injuries are listed in Table 3. The shoulder was the most injured body site (20%), with the next two most common being the leg (13%) and knee (11%). If the upper limb and lower limb data were each aggregated, the upper limbs (39%) comprised the sites of a similar number of injuries to those affecting the lower limbs (38%).

The natures of the injuries are shown in Table 4. The most frequent nature of injury was sprains and strains (51%), followed by muscle, tendon, and nerve injuries (32%, *n* = 182). These two natures of injury (sprains and strains, and muscle tendon and nerve injuries) accounted for most reported injuries (83%), with the proportion of injuries represented by the next-most reported nature of injury, bruise, and graze (4%), being notably lower.

The reported mechanisms of injury are shown in Table 5. The most common mechanisms of injury, accounting for more than twice as many injuries as the next-most reported mechanism, was muscle stress with physical exercise (31%), and this was followed by repetitive stress or forceful movements to muscle or joints (15%).

The activities being undertaken when injuries occurred are shown in Table 6. “Unknown or other” activities were reported most often as the source of injury (45%). The top known activity recorded as the source of injuries, which far outweighed any other activity reported, was police training (38%). Police training accounted for more than twice the number of injuries associated with all other known activities combined.

## 4. Discussion

The aim of this study was to profile the injuries sustained by NZ police trainees undergoing training, to inform future risk management strategies. The overall incidence rate of injuries over the assessed period for the law enforcement officers (LEO) trainees was 1550 injuries per 1000 person-years and ranged from 456 to 3079 injuries per 1000 person-years across the different 3-month time periods observed. A review of injuries in law enforcement by Lyons et al. [7] found the reported incidence of injuries among law enforcement personnel varied from 240 [27] to 2500 [28] injuries per 1000 person-years. Variations in the reported injury incidence rates between articles reported in the review [7] could, in many instances, be attributed to research design factors such as the included injury types, natures of the studies being compared, sources of data, and collection procedures (e.g., self-reported, workplace databases, etc.) and injury definitions employed [29]. Only three of the studies in this review reported incidence rates over 1000 injuries per person-years, being 1680 (Nabeel et al. [19]), 2320 (Sullivan and Shimizu [30]), and 2500 (Cho et al. [28]) injuries per 1000 person-years. It should be noted, however, that these injury rates were observed in fully qualified officers on duty, a population which differs from trainees.

As new police trainees are typically recruited from the general population, with varying levels of physical activity experience and fitness, the physical requirements of police training may exceed the trainee’s previous training loads and current capabilities. Therefore, it is unsurprising than new trainees are at a higher risk of injury when compared to their trained counterparts, with research suggesting that new military trainees have a high potential for injury due to the sudden increase in physical conditioning requirements, the complexity of new physical tasks, reduced opportunity for recovery, and resulting increased risk of overtraining [14,15,17]. Considering this, it is noteworthy that pre-enlistment fitness test standards for the New Zealand Police Force were lowered in 18 December 2017 [31] and, as such, less-fit trainees may have entered training, with these trainees at a greater risk of injury. These factors may have contributed to the notably higher incidence of injuries over 2018–2019 (overall, 1861.99 injuries per 1000 person-years) when compared to a similar period from 2017 to 2018 (overall, 1133.71 injuries per 1000 person-years).

However, the injury incidence rate during training was seen to fluctuate between the three-month periods (Table 1). One reason for this variation across three-month periods may include how many trainees were admitted as part of the intakes over the three-month periods. A recruiting drive began in the NZ Police Force in 2017 and the number of trainees increased from 500 to 1040 trainees in 2018. Subsequently, changes to the physical training program may have occurred within this period and greater numbers of less-fit trainees from the limited applicant pool may have been enlisted to fill available training places. Any change to the training program would alter the total amount of physical activity or cumulative loading, affecting the risk of injury [11], and lower levels of trainee fitness would increase individual injury risks [32].

Another potential factor affecting injury rates could be the increase in numbers of female trainees in the second year for which data was provided, as evidence shows differences in injury rates often occur between male and female trainees, with female trainees typically injured at higher rates [17,33]. Knapik et al. [17] found that among Federal Bureau of Investigation trainees, the injury incidence for females was 42%, as opposed to 35% for males. Within Army military police training, a further study by Knapik et al. [33] found female trainees again had a higher rate of experiencing at least one injury during training (67%, as opposed to 34% in males). Therefore, any potential changes in police recruitment to increase the number of female trainees may have also affected the injury rates presented in this study. 

One other possible reason for injury rate variation across time periods could include the changes in weather conditions between the three-month periods, potentially changing training surfaces. Falls are a common cause of injury reported within military personnel, causing up to 10% of total lower limb injuries in the New Zealand Defence Force [34]. Similar rates of falls are seen within the Police Force, with Reichard and Jackson [35] finding falls caused 11% of law enforcement officers injuries, and this study indicated 9% of all injuries to LEO trainees were from slips, trips, or a fall from any height. Drier conditions have been shown to have reduced injuries on grass surfaces when compared to wet or slippery conditions in sport [36]. Sherrard et al. [21] discusses the potential link between the shoe, surface, and shoe–surface interface contributing to knee and ankle ligament injury in the Australian Defence Force. Therefore, the association between weather conditions and falls may be another explanation for the variation in injury rates across the observed three-month periods. This finding may explain the uncharacteristically high incidence of injury of 3079.69 injuries per 1000 person-years over the July–September 2017 period, which recorded lower than typical sunshine (75–89% of winter normal) and higher than typical rainfall (131% above normal in Wellington).

One of the main findings of this study was that the most prevalent location of injury within NZ Police trainees was the shoulder, accounting for 20% of all injuries (Table 3). This finding supports those of previous studies involving law enforcement personnel [7,28,37]. A review by Lyons et al. [7] found that the upper extremity was the most common site of injury sustained in law enforcement officers, representing 33% to 43% of all musculoskeletal injuries, a finding similar to the 39% found in this study. Conversely, Larsen et al. [37] found injuries to the upper limb were 2.5 times more prevalent than injuries to the lower limb, and that injuries specific to the shoulder represented a total of 8% of the injuries within an Australian Specialist Policing Unit. These results differ to the findings of this study, which suggest very similar rates of injury between upper and lower limbs. A potential reason for this difference arises from the notably different law enforcement populations of these two studies. As opposed to the initial general duties police of this study, the study by Larsen et al. [37] investigated injuries in specialist police, who are known to carry heavier loads [38], and in this specialist population, shoulder strength is more strongly correlated to their load carriage performance than lower limb strength [39].

Despite similarities to reports in other policing populations, the results of the current study differ from those from tactical populations other than law enforcement. Unlike in law enforcement officers, the lower limb is most prevalent site of injury within military personnel, including those undertaking initial training [21,40,41,42,43,44,45]. In a study by Schram et al. [23], investigating injuries in Australian Army personnel undertaking basic training, the knee, ankle, lower leg, and foot were the most prevalent body sites, representing 13%, 11%, 10%, and 10% of all injuries, respectively. Of note, when compared to results of this study, proportions of injuries affecting the knee and lower leg were similar (11% and 13% respectively), whereas the proportions affecting the ankle and foot in the current study were notably lower (7% and 3%, respectively). As such, the results suggest that the major difference observed in distributions of lower limb presentations in the trainee LEOs, when compared to the Army trainees described by Schram et al. [23], may be due to the lower rates of ankle and foot injuries for the LEO trainees, while proportions of injuries that affect the leg and knee may be similar. This suggestion is supported by a study of injuries in US military personnel [43]. Hauret et al. [43] found a combined total of 22% of all injuries affected the knee or lower leg, results similar to those of Schram et al. [23], and this study (25% of injuries affected the leg and knee combined, Table 3).

Differences in the most common injury sites between law enforcement populations and the military, and particularly differences in the proportions of injuries that affect the foot and ankle, may be due to occupational task differences. These include differences in load carriage requirements. Police personnel are required to carry body armour and other accessories on their duty belts [46], adding an additional 8–10 kg [47,48]. However, military personnel load carriage often exceeds 45 kg [25,49]. Load carriage injuries are often overuse injuries [25,29,42,44,50]. Orr et al. [25] found that 56% of the reported load carriage injuries in the Australian Army were in the lower limb. Furthermore, foot injuries associated with load carriage tasks were more common in female soldiers and ankle injuries were more common in male soldiers [51].

Police trainees may also perform a higher volume of defensive tactics, including arm bars, locks, and similar, all of which place strain across the shoulder, not only for the trainee performing the role of arresting officer but also for the trainees acting as the perpetrator. The loads placed through the upper limbs may be further exacerbated by the lower levels of defensive tactics skills trainees possess when performing tasks, in comparison to trained instructors. The chronic, though lighter, loads that police are required to carry, and amount of defensive tactics undertaken, may explain why the results for LEO trainees in this study did not match those of military personnel, with the shoulder being the most common injury site for LEO trainees.

The most common nature of injury in this study was strains and sprains. This is in agreement with the review of Lyons et al. [7], who found the proportions of all injuries to LEO that were strains and sprains ranged from 42 to 95%. A study by Knapik et al. [17], investigating injuries among Federal Bureau of Investigation new agent trainees, found that strains and sprains were the most common types of traumatic new injuries, accounting for 27% of such injuries. Larsen et al. [37] investigated the injury profile of Australian Specialist Police and found that sprains and strains were the most common nature of injury, accounting for 61% of total injuries. Similar findings are reported in the military population, with a study on lower limb injuries in the New Zealand Defence Force finding strains and sprains of the ankle and knee represented 51% of total injuries [34], while a study of injuries in Australian Army soldiers found strains and sprains to represent 60% of minor personal injuries [52]. As such, the proportions of injuries that were observed to be sprains and strains in this police population present as consistent with not only other police services, but military services as well.

Muscle stress with physical exercise was the most common mechanism of injury. This result is similar to those from other studies investigating injury profiles in law enforcement populations. While in previous studies it is noted that the most common injury within active service law enforcement officers, as opposed to trainees, was noncompliant offenders/assault [19,37], training activity still accounted for 33% of all injuries [37]. Furthermore, this study’s finding that 31% of all injuries in the LEO trainee population were due to muscle stress with physical exercise (Table 5) is similar to findings in military trainees, with muscle stress with no objects being handled representing 41% of all injuries in the Australian Regular Army and 36% in the Reserve Army. [23] These results were similar in qualified Australian Army soldiers, where McDonald et al. [45] reported that muscular stress while lifting, carrying, or donning equipment accounted for 32% of total injuries. On this basis, given that the police trainees in this study were less likely than trained LEO to actively engage an offender and be subject to assault, this mechanism of injury is nevertheless almost as common in the LEO trainees as in qualified police officers and trainees and trained personnel of a military service.

In the LEO trainees from the current study, the most common activity when the injury occurred was reported to be “unknown or other” (45.4%). An injury is often reported as arising from an unknown activity when there is an insidious onset, with a specific activity not able to be determined as the cause of the injury. This may indicate an overuse injury, which occur commonly in law enforcement and military populations alike [17,53]. Schram et al. [23] found that during basic training within the Australian Army, an unknown activity accounted for 10% and 11% of the injuries reported within the Regular Army and the Reserve Army, respectively. While these results for LEO trainees may suggest a potentially high number of overuse injuries, it is possible that injury reporting and documenting practices may also have led to this outcome, given concerns noted with injury reporting in other tactical training populations [54].

The second-most common reported activity being undertaken when injury occurred in the LEO trainees was police training, representing 38% of injuries (Table 5). This finding is similar to results of other studies suggesting that, due to the rapid increase in physical demands and the high levels of exertion required for the various tasks, trainees undergoing training have more injuries than their trained counterparts [11,17,18]. Injury rates in trainees undertaking basic training may be as high as three times those recorded during post-training service within the military [32] and, similarly, the injury rate within the New Zealand Military trainees was found to be more than five times higher than that observed in trained personnel [34]. Within the FBI new trainees, the two most common new training injury cases arose from defensive tactics training and physical fitness training, contributing 58% and 20% of all injuries, respectively [17]. In the same study, injuries caused by physical fitness testing contributed 5% of new injuries, similar to this study’s finding of 5%. Within the Australian Army, combat training and physical training contributed 13% and 24% of all injuries, respectively (totalling 37%) [45].

One key limitation of this study is the potential for underreporting. Underreporting occurs when the reporting threshold may be high, so the numbers of injuries reported do not represent the real occurrence rates [54]. However, a benefit of the data collected in this study was that it came from point-of-care reporting, as opposed to retrospective self-reporting [54]. Point-of-care reporting has been shown to have a better rate of capture of injuries that have occurred and therefore to be more representative of the actual injury rate, as reported by Pope and Orr in the Australian Army context [54]. A final limitation of the current study was the lack of more detailed population data. While the number of trainees was available for specific training periods, the sex and age distributions of trainees in these periods was not available to inform analyses.

## 5. Conclusions

The shoulder was the most injured body site in the LEO trainees, accounting for 20% of injuries. The most prevalent nature of injury was sprains and strains, representing 51% of injuries. Muscular stress with physical exercise was the most common mechanism of injury, accounting for 31% of injuries. Injury minimization programs should target the shoulder in particular, with a specific focus on police training practices and the annual period of July to October. However, the lower limbs, notably the leg and knee, should not be ignored. Injuries appear to be often joint-related and cumulative in nature, without a known activity causing them. Prescreening protocols (e.g., history of previous injuries, movement ability, and fitness levels) may be of benefit, and efforts should be made to recruit and train physically resilient trainees and ensure that any injuries, be they pre-enlistment or occurring during training, are fully rehabilitated prior to trainee commencement as a qualified officer. Physical training loads, be it from physical training sessions or other physically demanding activities (e.g., defensive tactics), should be monitored and optimized to ensure performance gains and reduction of injury risks due to cumulative load on trainees. 

## Figures and Tables

**Table 1:** Numbers of trainee and trainee injuries during each three-month period of training.

Date Period	Number of Trainees	Number of Injuries (*n*)	% of Total Injuries	Injuries Per 1000 Person-Years
21/04/17–20/07/17	120	41	7.3%	1558.85 †
21/07/17–20/10/17	120	81	14.4%	3079.69 ‡
21/10/17–20/01/18	420	42	7.4%	456.25 ~
21/01/18–20/04/18	160	86	15.2%	2452.34 ‡
21/04/18–20/07/18	220	114	20.2%	2364.20 ‡
21/07/18–20/10/18	340	85	15.1%	1140.63 †
21/10/18–20/01/19	180	103	18.3%	2610.76 ‡
21/01/19–03/02/19 *	100	12	2.1%	547.50
Total	1660	564	100%	1550.15

* Last three-month period only included approximately one month of data: † significantly different (*p* < 0.001) from ‡,~: ‡ significantly different (*p* < 0.001) from †,~: ~significantly different (*p* < 0.001) from †, ‡.

**Table 2:** Ages of trainees with reported injuries.

Age (Years)	*N* (Injuries)	% of All Injuries
<20	11	2.0%
20–29	337	59.8%
30–39	165	29.3%
40–49	49	8.7%
50+	2	0.4%
Total	564	100%

**Table 3:** Body sites of injures in the NZ Police Force. Reported as number of injuries (percentage).

Location of Injury	
Shoulder	113 (20.0%)
Leg	75 (13.3%)
Knee	63 (11.2%)
Back	61 (10.8%)
Arm	44 (7.8%)
Ankle	41 (7.3%)
Wrist	41 (7.3%)
Neck	31 (5.5%)
Hip/groin	16 (2.8%)
Foot	16 (2.8%)
Hand	15 (2.7%)
Head	12 (2.1%)
Fingers/thumb	9 (1.6%)
Unknown	7 (1.2%)
Abdomen	6 (1.1%)
Chest	5 (0.9%)
Face	5 (0.9%)
Toe	4 (0.7%)
Total	564 (100%)

**Table 4:** Nature of injuries in the NZ Police Force. Reported as number of injuries (percentage).

Nature of Injury	
Sprain/strain	287 (50.9%)
Muscle/tendon/nerve	182 (32.3%)
Bruise/graze	23 (4.1%)
Other/unknown	21 (3.7%)
Fracture/dislocation/broken bone	17 (3.0%)
Contusion/crush	16 (2.8%)
Burn/radiation	11 (2.0%)
Superficial	3 (0.5%)
Puncture wound	2 (0.4%)
Skin disease	1 (0.2%)
Foreign object	1 (0.2%)
Total	564 (100%)

**Table 5:** Mechanism of injury in the NZ Police Force. Reported as number of injuries (percentage).

Mechanism of Injury	
Muscle stress with physical exercise	175 (31.0%)
Repetitive stress or forceful movements to muscle or joints	85 (15.1%)
Muscle stress—lifting or handling people or objects	69 (12.2%)
Hitting an object, animal, or person	65 (11.5%)
Slip, trip, or fall from same height	43 (7.6%)
Unknown/other	40 (7.1%)
Hit or trapped by an object, animal, or person	31 (5.5%)
Muscle stress with no objects (intrinsic)	31 (5.5%)
Contact with hot or sharp object	14 (2.5%)
Fall from height	10 (1.8%)
Contact with biological agent	1 (0.2%)
Total	564 (100%)

**Table 6:** Activity when injury occurred in the NZ Police Force. Reported as number of injuries (percentage).

Activity	
Unknown/other	256 (45.4%)
Police training	215 (38.1%)
Physical competency test	29 (5.1%)
Walking/running	29 (5.1%)
Sports injury in duty	17 (3.0%)
Lifting/moving/carrying object	10 (1.8%)
Police duties	7 (1.2%)
Motor vehicle accident	1 (0.2%)
Total	564 (100%)

## Data Availability

As this data is from a law enforcement agency it is only available though a reach forward request from the corresponding author to the agency.
